# Editorial: The impact of exposure to environmental chemicals, pharmaceuticals and particles via human breast milk: a focus on health effects and underlying mechanisms

**DOI:** 10.3389/fpubh.2025.1585347

**Published:** 2025-04-24

**Authors:** Charlotte Cosemans, Eline Tommelein, Michelle Plusquin

**Affiliations:** ^1^Centre for Environmental Sciences, Hasselt University, Diepenbeek, Belgium; ^2^Department of Pharmaceutical Chemistry, Drug Analysis and Drug Information, Center for Neurosciences, Research Group Experimental Pharmacology, Vrije Universiteit Brussel, Jette, Belgium

**Keywords:** breastfeeding, environmental pollutants, pharmaceuticals, human milk (HM), particles

## Introduction

Breastfeeding is widely recognized as the optimal source of nutrition for ensuring the health and wellbeing of infants, providing essential nutrients and antibodies that strengthen their immune system and support neurodevelopment (1–3). However, the presence of environmental pollutants and pharmaceuticals in human milk could raise concerns regarding potential adverse health effects on nursing infants, as it has been shown that both breastfeeding mothers and healthcare providers are concerned about the transfer of these exogenous substances in milk (4). This Research Topic aimed to explore the impact of exposure to various environmental chemicals, pharmaceuticals, and particles through human milk, focusing on understanding their biological effects on children and adolescents. The accepted studies in this Research Topic comprise a mini review, two brief research reports, and four original research papers, providing insights into the complexities of maternal transfer of these substances and emphasizing the need for continued research and funding to safeguard maternal and infant health.

## Contribution to the field

Naspolini et al. investigated the relation between metals (i.e., As, Pb, Hg, and Cd) in human milk and neurodevelopmental outcomes in infants between three and 16 months old, assessed with the Bayley-Scales of Infant and Toddler Development (Bayley-III). All metals, except Cd, were detected in breast milk and infants exposed to Pb had lower language development trajectories than non-exposed infants.

Five studies focused on pharmaceuticals in human milk. The review by De Hondt et al. examined the transfer of Attention Deficit/Hyperactivity Disorder (ADHD) medications into human milk and their potential impact on nursing infants. This review highlights the lack of methods to monitor multiple ADHD medications and the limited research on their quantification. This article emphasizes the need for further research to better understand medication transfer and develop clinical guidelines for mothers with ADHD during lactation. The case report by Van Neste et al. examined the transfer of monomethyl fumarate (MMF), the active metabolite of dimethyl fumarate (DMF), into human milk in a postpartum woman treated for multiple sclerosis. MMF concentrations varied widely in milk samples, but estimated infant exposure remained low, with relative infant doses (RIDs) between 0.16% and 0.22%. Combined with two previous cases (5), these findings suggest minimal MMF transfer to breast milk, though further research is needed to assess infant exposure and safety. Falconi et al. assessed the transfer of sacubitril/valsartan into human milk and the potential risk to infants. Valsartan was undetectable in all milk samples, while sacubitril and its active metabolite LBQ657 were present at low concentrations, with a combined RID of < 0.25%, well below the safety threshold of 10%. These findings suggest minimal exposure through breastfeeding, indicating a low risk to infants. Naidoo et al. investigated the impact of prenatal exposure to tenofovir diphosphate (TFV-DP) containing pre-exposure prophylaxis (PrEP) on growth metrics in HIV-unexposed, breastfed African infants and found no significant differences in weight, length, or head circumference Z-scores over 18 months. These findings suggest that prenatal exposure to TFV-DP does not affect early childhood growth. While previous studies specifically examine pharmaceuticals in human milk, mothers can be worried about the transfer of vaccine particles into milk. Despite a systematic review reporting no severe COVID-19 vaccine-related adverse effects in either mothers or nursing infants and no significant transfer of vaccine components into breast milk (6), vaccine hesitancy among breastfeeding women seems to remain high. Simsekoglu et al. examined COVID-19 vaccine hesitancy and attitudes among pregnant and breastfeeding women in Turkey. While participants showed a high level of vaccine hesitation, their overall attitude toward vaccination was positive. Factors such as working status, influenza vaccination history, smoking, and chronic disease influenced hesitancy and attitudes, highlighting the need for targeted strategies to address concerns in future pandemics (7).

Lastly, one study focused on environmental particles in human milk. Cosemans et al. showed for the first time that traffic-related particulate matter, such as black carbon particles, was detected in human milk and found an association with ambient air pollution concentrations at the maternal residential address. This study reported a novel pathway through which these particles can directly enter the infant's system, in addition to being exposed via inhalation. It has been previously established that prenatal exposure to particulate matter is linked to health outcomes in children, such as neurodevelopment (8), kidney function (9), and growth trajectories (10). However, the impact of this new route of exposure via human milk remains unknown and needs further investigation.

## Conclusion

The findings presented in this Research Topic underscore the necessity for heightened awareness and further investigation into the presence and effects of environmental pollutants and pharmaceuticals in human milk. Regarding pharmaceuticals, the studies highlighted methodological challenges, and for certain pharmaceuticals, a low level of exposure was detected. In contrast, in the context of environmental exposures, the detection of environmental particles in human milk presents entirely new challenges that warrant further investigation. Understanding exposure pathways and their implications for infant health is essential for developing effective public health policies and clinical guidelines. By continuing to explore these critical issues, we can alleviate any concerns families may have regarding the safety of their infants.

## References

[1] Cosemans C Nawrot TS Janssen BG Vriens A Smeets K Baeyens W. Breastfeeding predicts blood mitochondrial DNA content in adolescents. Sci Rep. (2020) 10:387. 10.1038/s41598-019-57276-z 31941967 PMC6962168

[2] Horta BL Victora CG. Long-Term Effects of Breastfeeding: a Systematic Review. (2013). Available online at: https://iris.who.int/handle/10665/79198

[3] Horta BL de Lima NP. Breastfeeding and type 2 diabetes: systematic review and meta-analysis. Curr Diab Rep. (2019) 19:1. 10.1007/s11892-019-1121-x 30637535

[4] De Hondt L Gorsen SL Verburgh P De Paepe K Muyldermans J Tommelein E. Health care providers' perspective and knowledge about peri-surgical medication and practices in breastfeeding women. Int J Environ Res Public Health. (2023) 20:3379. 10.3390/ijerph20043379 36834074 PMC9964632

[5] Ciplea AI Datta P Rewers-Felkins K Baker T Gold R Hale TW. Dimethyl fumarate transfer into human milk. Ther Adv Neurol Disord. (2020) 13:1756286420968414. 10.1177/1756286420968414 33193814 PMC7607748

[6] Muyldermans J De Weerdt L De Brabandere L Maertens K Tommelein E. The effects of COVID-19 vaccination on lactating women: a systematic review of the literature. Front Immunol. (2022) 13:852928. 10.3389/fimmu.2022.852928 35464406 PMC9024041

[7] Abu-Raya B Madhi SA Omer SB Amirthalingam G Giles ML Flanagan KL. Global perspectives on immunization against SARS-CoV-2 during pregnancy and priorities for future research: an international consensus paper from the world association of infectious diseases and immunological disorders. Front Immunol. (2021) 12:808064. 10.3389/fimmu.2021.808064 35003137 PMC8733958

[8] Cosemans C Madhloum N Sleurs H Alfano R Verheyen L Wang C. Prenatal particulate matter exposure is linked with neurobehavioural development in early life. Environm Res. (2024) 252:118879. 10.1016/j.envres.2024.118879 38579996

[9] Rasking L Van Pee T Vangeneugden M Renaers E Wang C Penders J. Newborn glomerular function and gestational particulate air pollution. EBioMedicine. (2024) 107:105253. 10.1016/j.ebiom.2024.105253 39178748 PMC11388157

[10] Cosemans C Wang C Alfano R Martens DS Sleurs H Dockx Y. In utero particulate matter exposure in association with newborn mitochondrial ND4L(10550A>G) heteroplasmy and its role in overweight during early childhood. Environm Health. (2022) 21:88. 10.1186/s12940-022-00899-z 36117180 PMC9484069

